# Recurrence Plot and Machine Learning for Signal Quality Assessment of Photoplethysmogram in Mobile Environment

**DOI:** 10.3390/s21062188

**Published:** 2021-03-20

**Authors:** Donggeun Roh, Hangsik Shin

**Affiliations:** Department of Biomedical Engineering, Chonnam National University, 50 Daehak-ro, Yeosu 59626, Korea; nodg0426@gmail.com

**Keywords:** convolutional neural network, mobile healthcare, photoplethysmogram, recurrence plot, signal quality assessment

## Abstract

The purpose of this study was to develop a machine learning model that could accurately evaluate the quality of a photoplethysmogram based on the shape of the photoplethysmogram and the phase relevance in a pulsatile waveform without requiring complicated pre-processing. Photoplethysmograms were recorded for 76 participants (5 min for each participant). All recorded photoplethysmograms were segmented for each beat to obtain a total of 49,561 pulsatile segments. These pulsatile segments were manually labeled as ‘good’ and ‘poor’ classes and converted to a two-dimensional phase space trajectory image using a recurrence plot. The classification model was implemented using a convolutional neural network with a two-layer structure. As a result, the proposed model correctly classified 48,827 segments out of 49,561 segments and misclassified 734 segments, showing a balanced accuracy of 0.975. Sensitivity, specificity, and positive predictive values of the developed model for the test dataset with a ‘poor’ class classification were 0.964, 0.987, and 0.848, respectively. The area under the curve was 0.994. The convolutional neural network model with recurrence plot as input proposed in this study can be used for signal quality assessment as a generalized model with high accuracy through data expansion. It has an advantage in that it does not require complicated pre-processing or a feature detection process.

## 1. Introduction

Photoplethysmography (PPG) is a technology that can acquire biometric information non-invasively using the property of light transmitting and reflecting through the human body. It has been widely used clinically for oxygen saturation and pulse rate measurement [1]. In addition, PPG is a promising bio-signal actively studied for cardiac output estimation [2], vascular stiffness assessment [3], cuffless blood pressure estimation [4,5,6], and pain assessment [7,8]. The use of PPG is increasing because it can provide a wealth of physiological information despite its simple measurement principle, simplicity of hardware configuration, and low cost. In recent years, with the increase of wearable healthcare and mobile healthcare, the number of devices equipped with PPG is also increasing. Indeed, it is no longer special for a wearable device to mount a PPG to provide functions such as measuring pulse rate, leading to the possibility of acquiring physiological information at any time in daily life. The biggest limitation to the use of PPG is its vulnerability to noise. In particular, motion artifact or low perfusion are the most significant noises that can distort the PPG waveform. This noise sometimes completely destroys the PPG waveform or makes it impossible to find pulsations, leading to incorrect information, misdiagnosis, and critical medical problems [1]. Various techniques have been proposed to solve such motion artifact or the low perfusion problem of PPG. However, there is no generalized method that can mitigate noise or restore the waveform because noise and PPG have similar time–frequency characteristics, making it difficult to remove noise when the signal is weakened due to physiological changes. This problem is becoming more serious due to increasing use of PPG in a mobile environment. Therefore, a method that can decrease errors in PPG analysis and screening sections in which a signal is distorted is required. Using such a method, not all information of the signal should be included in the analysis. Considering that PPG analysis result is mainly provided by a representative value such as an average value extracted from a large number of beats, such a method can increase the accuracy of the analysis by removing outliers.

With such a method, the most important thing is signal quality assessment. To assess signal quality, methods of comparing characteristics of PPG waveforms, methods based on template matching, and methods using machine learning have been proposed. Among these methods, the method of evaluating signal quality through characteristics of the waveform is based on the observation of signal features such as changes in amplitude, time interval, and feature point of PPG to evaluate signal quality using specific criteria. In a previous study, amplitude, pulse rising time, pulse-to-pulse interval, and number of diastolic peaks have been used as PPG characteristics for signal quality assessment [9]. In another study, amplitude, pulse-to-pulse interval, pulse width, ensemble mean, and Euclidean distance of all pulses have been used [10]. Kurtosis and Shannon entropy [11], and zero crossing rate [12] have also been proposed as signal quality assessment indicators. In a recent study, Song et al. [13] have proposed a signal quality index that includes effects of high-frequency noise, baseline change, and motion artifact. As a method for assessing signal quality based on PPG template, waveform quality has been evaluated through adaptive template matching after generating an ensembled average waveform of the entire beat [14]. Such a template-based waveform quality assessment method mainly creates a template for a high-quality normal pulse and obtains a correlation coefficient between the normal template and each pulse waveform to distinguish between normal and abnormal waveforms. In signal quality assessment through template matching, a dynamic time warping method is often used to equalize the pulse width of a PPG pulsation [15,16]. In addition to the signal quality assessment method using morphological characteristics or a template of PPG, machine learning-based signal quality assessment methods have also been actively studied in recent years. Liu et al. [17] have proposed a five-layer fuzzy neural network-based signal quality assessment algorithm to assess the signal quality of PPG and reported a sensitivity of 0.8–0.9. In another study, Liu et al. [18] have classified PPG quality into three levels (high, middle, and low) using PPG and differential PPG as inputs for a two-dimensional deep convolution neural network and residual deep convolutional neural network. Naeini et al. [19] have binarily classified PPG quality into ‘reliable’ and ‘unreliable’ using the entire 60-s PPG signal as a convolutional neural network (CNN) input without feature extraction.

As described above, various methods have been proposed to evaluate the signal quality of PPG. However, previous research results cannot be generalized due to the use of a small number of subjects. In particular, in the case of assessing signal quality based on features of waveforms, an error such as ‘circular reasoning’ might occur. This paradox is that the quality of the signal is ultimately assessed for the purpose of extracting features, although the feature must be extracted first before assessing signal quality. Therefore, a reliable signal quality evaluation method that does not depend on feature extraction is needed. Indeed, in the method of assessing the quality of a signal based on the waveform features, it is necessary to verify that the waveform features are well-extracted during the development stage, and for this, a manual verification procedure by an experienced expert is required. In this case, two manual verification procedures, waveform quality classification and feature point extraction, are required to accurately assess the quality of the signal, which significantly increases the complexity and cost of human resources. Also, since the error of the feature detector can affect the performance of the waveform quality assessment, a method that is not dependent on feature extraction is required.

In this study, we proposed a method of assessing the quality of PPG signal using only the original signal without using additional processed features for quality assessment of the PPG signal. The aforementioned studies showed that the quality of a PPG waveform can be assessed based on spatiotemporal features. Thus, we used spatiotemporal information of PPG by expanding a one-dimensional PPG waveform into a two-dimensional image using a PPG recurrence plot that can be exploited to characterize the system’s behavior in phase space, and assessed the signal quality using a convolutional neural network known to have strengths in image classification.

## 2. Materials and Methods

### 2.1. Dataset

PPG data were obtained from 76 subjects (29 males and 47 females) with a mean age of 52.3 ± 10.8 years, a mean height of 161.3 ± 9.0 cm, and a mean weight of 62.3 ± 13.3 kg. PPG was recorded on the left index finger in a supine position for 6 min at a frequency of 300 Hz using a Carescape Monitor B650 (GE Healthcare, Chicago, IL, USA). All data were obtained after obtaining approval from the Institutional Review Board of Asan Medical Center (Songpa-gu, Seoul, Republic of Korea). Written informed consent was obtained from all participants (IRB no.: 2016-0477). After detecting pulse onset as the starting point of a pulsation, measured PPG was segmented and pulse quality was manually labeled for each pulsation. An adaptive threshold peak detection method was used for pulse onset detection. Pulse quality labeling was performed by three skilled researchers. Pulse quality was defined as excellent, acceptable, unfit, and unusable. Classification criteria and waveform examples are shown in Table 1. These classified pulse segments were finally grouped into two classes (good’ and ‘poor’) for pulse quality evaluation. Excellent and acceptable pulse segments were grouped as the ‘good’ class while unfit and unusable pulse segments were grouped as the ‘poor’ class. As a result, the dataset was divided into 46,057 pulse segments of ‘good’ class and 3504 pulse segments of ‘poor’ class out of a total of 49,561 pulse segments. The number of pulse segments corresponding to pulse quality labels is shown in Table 2.

### 2.2. Recurrence Plot

A recurrence plot is a way to visualize the recurrence state in a phase space [20,21]. This method allows the representation of an m-dimensional phase space trajectory in two dimensions based on its recurrence. The recurrence between time i and time j is presented by a two-dimensional array. It has values of 1 and 0. Each dimension has a time unit. Recurrence between time i and time j is expressed with the following formula:

(1) $$R_{i,j}=\Theta (\varepsilon_{i}-∥{\bar{x}}_{i}-{\bar{x}}_{j}∥),{\bar{x}}_{i}\in {\mathbb{R}}^{m},\;i,\;j=1,\;\ldots ,\;N$$

where N is the number of states of $x_{i}$, $\varepsilon_{i}$ is the threshold distance, ∥·∥ is norm, and $\Theta \left(\cdot \right)\;$is the unit step function.

The recurrence plot is known as a useful method for analyzing linear dynamic systems with non-linear features. It can be used to analyze physiological rhythmic systems, including mechanical, electrical, chemical, neural, hormonal, and special activities [20]. For example, recurrence plots have been used to estimate paroxysmal atrial fibrillation based on the RR interval [22] and identify epileptic electroencephalogram [23]. Recently, a recurrence plot has been used to develop a computer-aided diagnosis system incorporating CNN known to be effective for two-dimensional image processing [24]. The recurrence plot algorithm expresses the distance between *m*-dimensional motion trajectories of one-dimensional time-series data as a two-dimensional matrix, allowing multidimensional machine learning structures such as CNN to be applied to one-dimensional data. In the present study, each segmented beat waveform was converted to a recurrence plot to generate a two-dimensional image as the input to CNN. Since the number of samples for each pulse segment is different, a recurrence plot was created from the pulse segment and then bicubic was interpolated to create an image of size 124 × 124. MATLAB^®^ 2018b platform (Mathworks, Natick, MA, USA) was used for all pre-processing, segmentation, and recurrence plot creation processes.

### 2.3. Convolutional Neural Network Model

**Figure 1:**
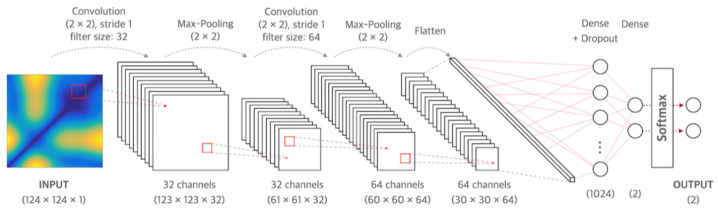
Architecture of the convolutional neural network used in this study.

**Figure 2:**
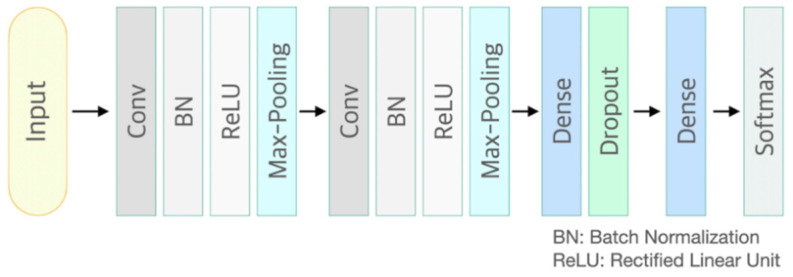
Functional structure of the proposed convolutional neural network architecture. Conv: convolutional neural network layer; BN: batch normalization layer; ReLU: rectified linear unit activation; Max-Pooling: max pooling layer; Dropout rate = 0.5.

CNN is a neural network that can be used to derive results from a convolution operation using a multidimensional kernel. It has the advantage of using features that reflect various dimensional features of data [25]. After winning AlexNet’s ILSVRC (ImageNet Large-Scale Visual Recognition Challenge) in 2012 [26], CNN has shown excellent results in the field of image recognition. The CNN model of the present study consisted of two convolutional layers and two fully connected layers. The first convolution layer contained 32 kernels of size 2 × 2 with stride 1. The second convolution layer contained 64 kernels of size 2 × 2 with stride 1. These two convolutional layers were followed by max pooling layers, the output of which went into a series of two fully connected layers through flattening. The second fully connected layer was fed into a Softmax layer with two class labels. The architecture of CNN proposed in this study is presented in Figure 1. The functional structure of the proposed CNN architecture is shown in Figure 2. Two convolutional layers with rectified linear unit (ReLU) activation [26] including batch normalization [27] and max-pooling layer were followed by two linear output layers with a softmax. The first dense layer included dropout (dropout rate = 0.5) [28], and the ReLU is applied as an activation function for all layers.

### 2.4. Model Development and Validation

A five-fold cross validation was used for model development and validation. In this five-fold cross validation, the dataset was divided into five sub-datasets with the same length. Four sub-datasets were used for model training. The rest of the sub-dataset was used to evaluate the trained model. This process was repeated five times so that all datasets could be used in the evaluation set once. Cross-validation has the advantage in that all datasets can be used evenly for training and evaluation. In the present study, to prevent overfitting when training the model and to improve versatility of the model, 25% of the development set was randomly extracted in every batch and used as a validation set. As a result, the data set was divided into 80% development set and 20% test set for each fold. The development set was further divided into 75% training set and 25% validation set. Eventually, 60%, 20%, and 20% of the total data for each fold were used as a training set, a validation set, and a test set, respectively. In the model development stage, training loss generally decreased. Validation loss also decreased initially with increasing epoch. However, it tended to increase after a specific training epoch. Since validation loss increased after a certain point, meaning that the model was overfitting, the model in the epoch with the minimum validation loss was selected as the model to minimize overfitting in the present study. Test accuracy was then evaluated. To evaluate the model’s performance, performance metrics such as accuracy, sensitivity, specific positive predictivity value and area under the curve were calculated. Accuracy is defined as the ratio of correctly classifying good- and poor-quality waveforms among all cases. Sensitivity refers to the ratio of the waveforms correctly classified as poor among the actual poor waveforms. Specificity refers to the ratio of the actual good quality waveforms that are correctly classified as good. The positive predictive value represents the proportion of waveforms that actually have poor quality among the waveforms classified as poor quality. The area under curve (AUC) is a binary classification metric, and is obtained through the area under the receiver operating character (ROC) curve, which is a curve that shows how the performance of the classification model changes as the threshold varies. AUC is frequently used for representing the effectiveness of the model. AUC has a 0 to 1 range, and the higher the AUC score, the better the model.

The class imbalance problem occurs when the class distributions are highly imbalanced. Many classification learning algorithms have low predictive accuracy for the infrequent class. Cost-sensitive learning is frequently used for solving this problem. In this research, the difference in the number of good and poor classes in our dataset can cause a class imbalance problem. Therefore, weight balancing that balances data by altering the weight that each training example carries when computing the loss was used for solving the problem of unbalanced datasets between ‘good’ class and ‘poor’ class during the model training. Class weight was calculated with the following formula—class weight = number of samples/(number of classes × number of samples by classes). The Adam (adaptive moment estimate) [29] is used for cost optimization in model development with 0.9 of exponential decay rate of the moving average gradient ($\beta_{1}$) and 0.999 of exponential decay rate of the moving average of the squared gradient ($\beta_{2})$. In the learning process, the learning rate was set to be 0.0001 and the batch size was set to be 8. The proposed CNN model was developed and validated with a 3.8 GHz Intel Core i7-8700 processor, 64 GB 1600 MHz DDR3 RAM, NVIDIA Geforce GTX 1080Ti, Python 3.6.7: Anaconda, Tensorflow 2.0.

## 3. Results

### 3.1. Recurrence Plot Analysis

**Figure 3:**
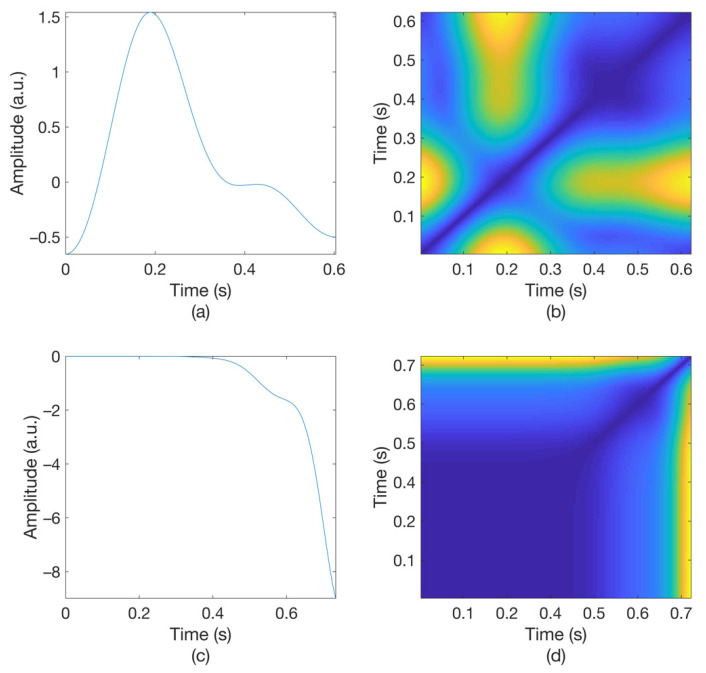
Examples of original waveform and the recurrence plot of photoplethysmogram segments. (**a**) Photoplethysmogram segment of ‘good’ class, (**b**) recurrence plot of ‘good’ class photoplethysmogram segment, (**c**) photoplethysmogram segment of ‘poor’ class, (**d**) recurrence plot of ‘poor’ class photoplethysmogram segment.

Figure 3 shows the original waveform and the recurrence plot of PPG segments classified as ‘good’ class (Figure 3a,c) or ‘poor’ class (Figure 3b,d). For ‘good’ class PPG segments (Figure 3a), a clear pulse onset, a systolic peak, and a dicrotic notch were observed. The pulse width was about 0.6 s. In the recurrence plot of ‘good’ class PPG segments (Figure 3b), the symmetric component corresponding to the systolic phase appeared in the lower left corner (0.1- to 0.3-s area). The component by diastolic phase including the dicrotic notch was shown in the upper right (0.3–0.6 s) section. In this way, it was confirmed that spatio-temporal characteristics in the systolic phase and the diastolic phase were expressed in the recurrence plot (RP) of normal PPG segments. Abnormal PPG segments showed a different RP pattern from normal PPG segments. For example, for signals that could not observe pulsating characteristics of PPG at all as shown in Figure 3c, the recurrence plot (Figure 3d) showed a completely different pattern from the recurrence plot of normal PPG segments (Figure 3b). In this case, the recurrence plot had a change pattern similar to the original PPG segment used for recurrence plot generation. Figure 3 shows examples of the PPG waveform and recurrence plot, including waveform (Figure 3a) and recurrence plot (Figure 3b) of a ‘good’ class PPG segments and waveform (Figure 3c) and recurrence plot (Figure 3d) of ‘poor’ class PPG segments.

### 3.2. Classification Results

**Figure 4:**
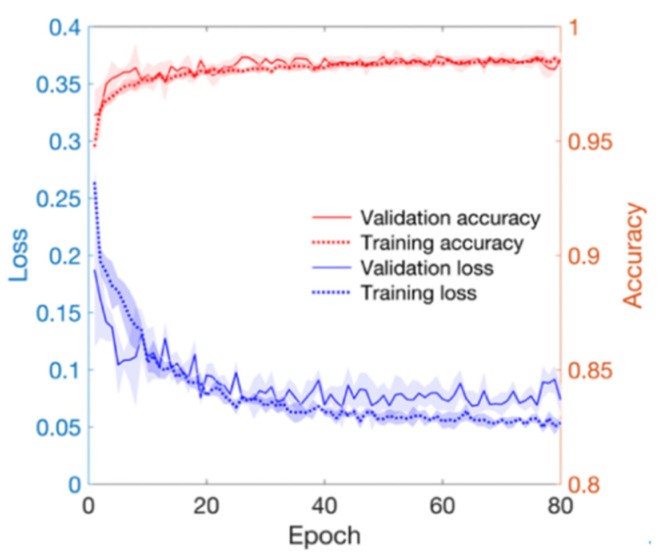
Loss and accuracy of training and validation processes. Red lines mean accuracy and blue lines mean loss.

**Figure 5:**
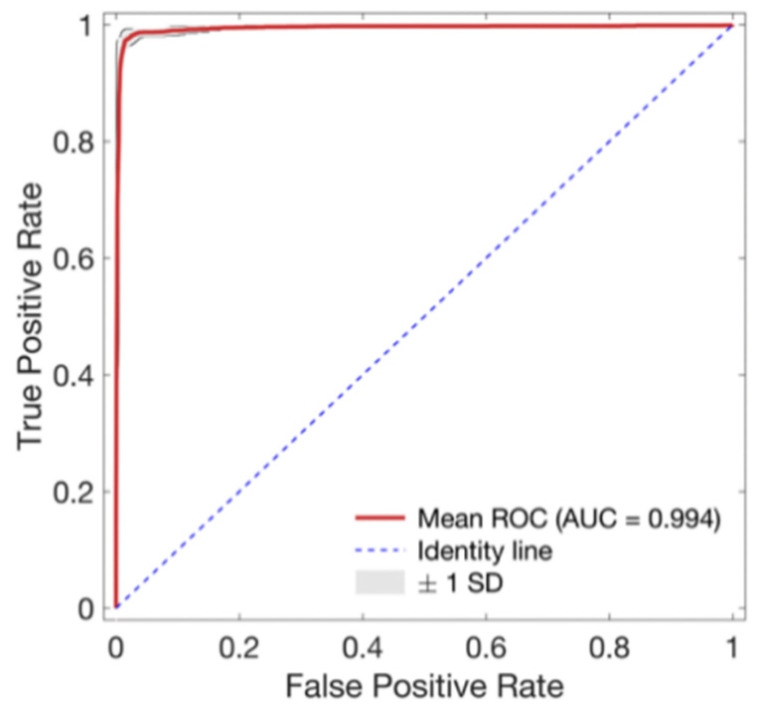
Receiver operating characteristic (ROC) curve for the proposed signal quality assessment model. Red line and gray area indicate mean ROC curves and ±1 standard deviation (SD) of ROC curves of every fold, respectively.

As training epoch increased during model training, loss reduction and accuracy increase were observed in both the training set and validation set (Figure 4). In Figure 4, the red line and the right axis represent average accuracy while the blue line and the left axis represent average loss. The solid line means results of the validation set. The dotted line means results of the training set. All results are expressed as average values due to cross validation. The shade means ± 1 standard deviation range for each value. Validation loss decreased with increasing training epoch. It then increased after around 60 epochs. Therefore, the epoch when the validation loss had the minimum value near 60 epochs was selected as the optimal epoch and the model was used for testing. Confusion matrix for the test set of the trained model is shown in Table 3. The developed model correctly classified 48,827 segments out of a total of 49,561 segments. It also miscalculated 734 segments, showing a balanced accuracy of 0.975. Its sensitivity, predictivity, and positive predictivity values for poor class detection were 0.964, 0.987, and 0.848, respectively. Table 4 shows its classification performances for training, validation, and test sets. Overall, it showed the best performance for the training set based on area under the curve (AUC = 0.998). It showed equivalent performance for the validation set and the test set (AUC = 0.994). Figure 5 shows mean and standard deviation range of the ROC curve of the developed model for the test set.

## 4. Discussion

The final purpose of signal quality assessment is to improve the accuracy of analysis results or to detect key information (e.g., feature points) of signals by defining signals of poor quality and excluding them from analysis. Therefore, the model for assessing signal quality inevitably involves receiving and analyzing signals of poor quality. In this case, an algorithm for assessing signal quality based on morphological features finally falls into the paradox that a technique used for reducing error of feature detection requires sophisticated feature detection. Also, as the quality of a signal degrades, the performance of signal quality assessment algorithm may also become lower. In the present study, a recurrence plot proposed as the input of the signal quality assessment model was used as CNN input by only changing the representation of the original signal from one-dimensional to two-dimensional, unlike previous models that analyze the input signal precisely and find features. There is an advantage in that an input feature robust against distortion of an input signal can be generated without a complicated or cumbersome signal pre-processing process including feature detection. In addition, it is possible to analyze distortion of a signal through spatiotemporal linkage of the waveform. This rationale is similar to the basis for determining general waveform distortion. The CNN classifier used in this study with recurrence plot inputs resulted in a very high (AUC = 0.994) test performance, similar to training performance (AUC = 0.998) and validation performance (AUC = 0.994). This means that the proposed model has a high level of generality. Compared to results of previous studies (see Table 5), waveform classification accuracy of both Sukor et al. [10] and Liu et al. [17] is 0.830, which is much lower than accuracy of proposed model (0.975 of accuracy). Moreover, in studies of Selvaraj et al. [11], Li and Clifford [15], Liu et al. [18], and Naeini et al. [19], the subject group was small (n < 15), therefore, the reliability of the model could be relatively low. Fischer et al. [9] showed the highest performance in previous studies, showing slightly higher (0.003 of accuracy) performance than ours. However, Fischer et al.’s research pre-supposes the detection of the features of the waveform, therefore it is dependent on the feature detector in practical use, and the computational complexity is high, and the trade-off between accuracy and convenience needs to be considered. Except for the method described by Liu et al. [18], most methods have complex pre-processing processes such as extracting features of the waveform for signal quality evaluation which involves the paradox that the feature is extracted by analyzing a distorted waveform that is difficult to detect and then determines whether to analyze the signal. These approaches may not be proper to perform signal quality assessment. In addition, the performance may be greatly affected by the quality of the original signal. On the other hand, the method proposed in this study can be applied to any signal regardless of signal distortion since it is used for quality evaluation by applying only dimensional transformation without any special processing of the original signal. The developed method showed somewhat low positive predictivity value mainly due to imbalance of the dataset. The ratio of ‘good’ dataset to ‘poor’ dataset used in this study was approximately 10:1 or higher. Degrading performance might have occurred due to an absolute small number of poor classes, although the training weight was adjusted and the dataset was stratified. Meanwhile, its performance is expected to be improved when more data are added and a balanced dataset is used.

## 5. Conclusions

We proposed the method of assessing waveform quality into ‘good’ and ‘poor’ classes by applying two-dimensional CNN after dimension expansion of one-dimensional PPG using the recurrence plot and verified that a performance of the proposed model is comparable to the results of previous research showing the highest level of performance. Noteworthy in this study is that the proposed model showed excellent performance without separated complex pre-processing and feature detection steps. In addition, the proposed CNN-based machine learning model showed generalized results without overfitting, showing the possibility to obtain robust results with a small dataset. However, in this study, the quality of the waveform is classified into only two grades, good or poor, and there is a limitation in that it cannot provide information on more sub-divided waveform quality. Therefore, in future research, it is necessary to develop an improved method to classify the waveform quality into multiple grades. In addition, in this study, although the PPG waveform morphology can be changed by various factors such as measurement body site, aging, vascular status, cardiovascular disease, this study did not include the quality assessment of the waveform deformed by these effects. Therefore, there is a need for this to be extended to research based on expanded data covering PPG of cardiovascular disease patients and various age groups.

## Figures and Tables

**Table 1:** Category, decision criteria, and waveform example for pulse quality labeling.

Class	Decision Criteria	Waveform
Excellent	Systolic phase with clearly distinguished and appropriate interval length Diastolic phase with one inflection point Distinct dicrotic features Pulse width in the range of 0.5–1.2 s	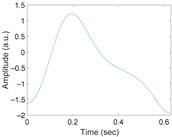
Acceptable	Systolic phase with clearly distinguished and appropriate interval length Diastolic phase with one inflection point Dicrotic notch that is distinguishable but not clear Pulse width in the range of 0.5–1.2 s	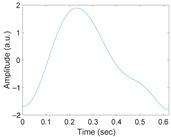
Unfit	Systolic phase with not clearly distinguished and inappropriate interval length Diastolic phase with multiple inflection points Indistinct dicrotic features Pulse width less than 0.5 s or more than 1.2 s	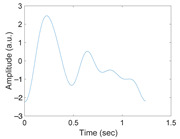
Unusable	Indistinguishable pulse shape No pulsation	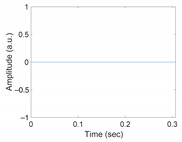

**Table 2:** Number of pulse segments corresponding to pulse quality labels.

Pulse Quality Label	Good		Poor		Total
	Excellent	Acceptable	Unfit	Unusable	
Number of Pulse Segments	14,644	31,413	3196	308	49,561
	46,057		3504		

**Table 3:** Confusion matrix.

		Estimated	
		Good	Poor
Actual	Good	45,449	608
	Poor	126	3378

**Table 4:** Classification performance of the proposed signal quality assessment model using training, validation, and test datasets.

Average Value of 5-Fold cross Validation	Dataset		
	Training (N = 29,737)	Validation (N = 9912)	Test (N = 9912)
Accuracy *	0.987	0.974	0.975
Sensitivity	0.990	0.977	0.964
Specificity	0.987	0.981	0.987
Positive predictivity value	0.870	0.866	0.848
Area under curve	0.998	0.994	0.994

* Accuracy means balanced accuracy.

**Table 5:** Signal quality assessment performance compared to previous studies (N: Number of subjects).

Reference	N	Sensitivity	Specificity	Positive Predictivity Value	Accuracy	Input
Proposed	76	0.964	0.987	0.848	0.975	Raw signal
Liu et al. [18]	14	0.920	0.920	0.960	0.950	
Naeini et al. [19]	1	0.830	-	0.830	-	
Fischer et al. [9]	69	0.994	0.920	0.984	0.978	Detected features
Sukor et al. [10]	13	0.890	0.770	-	0.830	
Selvaraj et al. [10]	10	0.993	0.938	-	0.948	
Li and Clifford [15]	13	-	-	-	0.952	
Liu et al. [17]	10	0.810	0.900	0.940	0.830	

## Data Availability

The data presented in this study are available on request from the corresponding author. The data are not publicly available due to data sharing statement is not included in initial project proposal.
